# Labor characteristics and intrapartum interventions in women with vaginal birth after cesarean section

**DOI:** 10.1186/s12884-022-04919-1

**Published:** 2022-07-23

**Authors:** Yehui Lan, Shuangjia Pan, Baoyi Chen, Lingli Peng, Ruyang Chen, Ying Hua, Yanyan Ma

**Affiliations:** 1 Department of Obstetrics and Gynecology, the Second Affiliated Hospital of Wenzhou Medical University, 325027 Wenzhou, China; 2 Department of Obstetrics and Gynecology, The Wenzhou Third Clinical Institute Affiliated to Wenzhou Medical University, Wenzhou, China; 3 Department of Obstetrics and Gynecology, The Dingli Clinical College of Wenzhou Medical University, Wenzhou, China

**Keywords:** Vaginal birth after cesarean section (VBAC), Labor characteristics, Intrapartum interventions, Perinatal outcomes

## Abstract

**Background:**

With the development of China’s two-child-policy, vaginal birth after cesarean section (VBAC) has aroused public concern. It is important to understand the labour characteristics and intrapartum management of women attempting VBAC to enhance the rates of successful VBAC. The purpose of our research was to investigate the differences in the characteristics of labor, intervention measures and perinatal outcomes between women who had a VBAC and primiparas or multiparas not undergoing VBAC, providing clinical references of intrapartum management for women who are planning a VBAC.

**Material and methods:**

This observational retrospective study enrolled all women who laboured spontaneously and who had a VBAC (*n* = 139) at the Second Affiliated Hospital of Wenzhou Medical University in China between 2016 and 2019. They were allocated into VBAC group A (the previous cesarean section was performed before dilation of the cervix) and VBAC group B (the previous cesarean section was performed after dilation of the cervix). The primipara control group included 149 primiparae, and the multipara control group included 155 multiparae with second vaginal birth. Durations of labor, intervention measures and perinatal outcomes were compared among the groups.

**Results:**

The durations of labor, intrapartum interventions and maternal and neonatal outcomes in VBAC group A were similar to those of the VBAC group B. However, all women who had a VBAC and those in VBAC group A had shorter first, second and the total stages of labor than primiparae. All women with VBAC and those in VBAC group B had longer second stage of labor, but shorter third stage of labor than multiparae. Oxytocin, labor analgesia and artificial rupture of membranes were administered less often in women with VBAC than in primiparae, while phloroglucinol was administered more often in women with VBAC than in multiparae. Women who had a VBAC were more likely to receive episiotomy and had higher incidences of postpartum hemorrhage than primipara and multipara women.

**Conclusions:**

Labor characteristics, intrapartum interventions and perinatal outcomes in women who had a VBAC with cervical dilation were similar to those in women who had a VBAC without cervical dilation before the previous cesarean section, but differed significantly from those of multiparae and primiparae who did not undergo VBAC.

**Supplementary Information:**

The online version contains supplementary material available at 10.1186/s12884-022-04919-1.

## Background

Cesarean section (CS) rate worldwide has increased from 10% to 21% over the past 15 years with a continuously increasing trend annually by 4% [1]. It’s worth noting that half of all unnecessary caesareans occur in Brazil and China [1]. This has aroused a significant global concern due to the economic burden and the increased complications after CS, especially in China after the implementation of “universal two-child policy” [2, 3]. Women following a CS opt to have a second child either by repeated cesarean section (RCS) or vaginal birth after cesarean section (VBAC) [3]. Due to the surgical indication of uterine scar, the phrase ‘once a cesarean, always a cesarean’ has always been repeatedly supported [2, 4].

However, some studies suggest that RCS does not appear to produce the benefits previously attributed to it [2, 5]. Actually, RCS increased respiratory morbidity, hospital costs and the length of hospital stay in neonates [6]. VBAC, as a relevant factor in decreasing overall CS rate, not only shows a lower incidence of postpartum hemorrhage (PPH), embolism disease and infection, but also offers advantages in terms of reducing health-care costs, enhancing patient satisfaction, and facilitating faster recovery from giving birth [4, 5, 7]. Therefore, VBAC should be encouraged in women with a prior low transverse cesarean birth rather than RCS [8].

Currently, most studies focus on the success factors and risks of planned VBAC, and the maternal and neonatal outcomes of VBAC. However, we know little about labour characteristics and intrapartum interventions. Therefore, in this study, we identified the differences in the characteristics of labor, intervention measures and perinatal outcomes between women who had a VBAC and primipara or multipara women who did not undergo VBAC, providing clinical evidence of labour management for women who are planning a VBAC.

## Methods

### Study design, population, sample and data collection

This is an observational retrospective cohort study including all women who had a VBAC and gave birth vaginally with spontaneous labor at the Second Affiliated Hospital of Wenzhou Medical University in China from January 2016 to December 2019. Pregnant women with VBAC were those who underwent a cesarean section with low-transverse segment at the time of their first birth and went into spontaneous labor at VBAC. The controls included primaipara and multipara control groups (women not undergoing VBAC) who were in accordance with the inclusion criteria followed and gave birth during the same period. These women were matched with women in the VBAC group on baseline characteristics including age composition, BMI, gestational age and newborn weight. The ratio of primipara and multipara controls to women in the VBAC group was 1:1, respectively.

Participants were eligible with singleton term pregnancy, cephalic presentation, the gestational age of 37-42 weeks, and without pregnancy complications including hypertension disorders, diabetes mellitus, intrahepatic cholestasis of pregnancy, placenta previa, placental abruption, oligohydramnios, and polyhydramnios. Exclusion criteria were women with prior classical, T-incision or longitudinal-incision, previous myomectomy, breech position, fetal birth weight of < 2.5 kg or > 4.0 kg, as well as their newborns with major congenital malformations (congenital anal atresia, congenital biliary atresia, congenital heart disease and so on). Women were excluded if any information on maternal characteristics, durations of labor, intervention measures or perinatal outcomes was not available. Women who had a VBAC were allocated into VBAC group A (the previous cesarean section was performed before dilation of the cervix, n=109) and VBAC group B (the previous cesarean section was performed after dilation of the cervix, *n*=30). The control group included 149 primipara women and 155 multipara women. We reviewed the labour processes of all eligible participants in the electronic medical record database. Baseline characteristics included maternal age, body mass index (BMI), gestational age and neonatal birth weight. The main outcomes were the duration of each stage of labor and the total labor, and intrapartum interventions including artificial rupture of membrane (AROM), phloroglucinol usage, oxytocin usage, labor analgesia, forceps delivery and mediolateral episiotomy, which were associated with the success rate of VBAC. Additionally, maternal and neonatal complications included Apgar scores at 1 min and 5 min, perineal laceration, postpartum urinary retention, blood loss during birth and 2 hours postpartum, PPH (defined as the blood loss of more than 500 ml following a vaginal birth), and the incidence of fever (T >38 ℃ during labor).

Intrapartum interventions were selected according to the process of labor. For example, artificial membrane rupture and oxytocin were used to accelerate labor by enhancing the uterine contractions. Phloroglucinol has a softening effect on cervical tissue and can coordinate uterine contractions and relieve pain, thus promoting labor [9, 10], which was recommended in our institution. Epidural anesthesia was encouraged to provide pain control. Forceps were used for operative vaginal birth for maternal and fetal indications. Mediolateral episiotomy was advised if necessary.

### Statistical analysis

SPSS 22.0 software was used for statistical analysis. The comparisons of continuous variables with normal distributions (mean ± standard deviation) were conducted by Student t-test. The comparisons of continuous variables without normal distribution (median with interquartile range) were conducted by Mann-Whitney U test. The comparisons of categorical variables (percentage) were conducted by Pearson’s Chi-square test or Fisher exact probability test. All *P*-values were two-sided and if below 0.05 the results were considered statistically significant.

### Results

During the study period, a total of 443 women were enrolled and included in the final analysis. These women were categorized into two groups: women with VBAC (total VBAC group, *n*= 139) and women who had a vaginal birth but not undergoing VBAC (control group, *n*= 304). The total VBAC group was divided into two subgroups: the VBAC group A (women who had a VBAC with cervix dilation before the previous CS, *n*=109) and the VBAC group B (women who had a VBAC without cervix dilation before the previous CS, *n*=30). The control group included 149 primipara women and 155 multipara women not undergoing VBAC.

### Baseline characteristics of the study groups

As shown in Table 1, there were no significant differences in demographic characteristics among the groups (*P* > 0.05).

**Table 1:** Baseline characteristics of the study groups

	total VBAC group (*n* = 139)	VBAC group A (*n* = 109)	VBAC group B (*n* = 30)	Primipara Control group (*n* = 149)	Multipara Control group (*n* = 155)	*P* values				
						*p*^*a*^	*p*^*b*^	*p*^*c*^	*p*^*d*^	*p*^*e*^
Mother’s age (years)	30.36 ± 3.76	30.21 ± 3.75	30.90 ± 3.81	29.87 ± 2.68	29.85 ± 3.29	0.198	0.217	0.389	0.121	0.376
Antepartum BMI (kg/*m*^2^)	25.69 ± 2.68	25.49 ± 2.69	26.41 ± 2.55	25.26 ± 2.74	25.56 ± 2.60	0.188	0.689	0.515	0.104	0.097
Gestational age (weeks)	38.82 ± 0.97	38.85 ± 0.97	38.69 ± 0.98	38.88 ± 1.08	39.01 ± 0.87	0.623	0.073	0.749	0.075	0.433
Newborn weight (g)	3225.76 ± 369.32	3200.18 ± 357.26	3318.67 ± 402.93	3213.02 ± 285.27	3295.87 ± 283.63	0.743	0.067	0.845	0.709	0.120

The comparisons of baseline characteristics of the study groups were with normal. distributions and were conducted by Student t-test

Data are given as mean ± SD

^a^ The comparison between total VBAC group and Primipara Control group

^b^ The comparison between total VBAC group and Multipara Control group

^c^ The comparison between VBAC group A and Primipara Control group

^d^ The comparison between VBAC group B and Multipara Control group

^e^ The comparison between VBAC group A and VBAC group B

### The duration of labor

There were no significant differences in the duration of all stages of labor between VBAC group A and VBAC group B (*P* > 0.05). Compared with women in the primipara control group, women in the total VBAC group and VBAC group A had shorter first, second and the total stage of labor (*P* < 0.05) (Table 2). In contrast, compared with women in the multipara control group, women in the total VBAC group and VBAC group B had longer second stage of labor and shorter third stage of labor (*P* < 0.05). No differences were found in the first and the total stage of labor between the total VBAC group and the multipara control group, as well as between the VBAC group B and the multipara control group (*P* > 0.05). There was also no difference in the duration of the third stage of labor between the total VBAC group and the primipara control group, or between VBAC group A and the primipara control group (*P* > 0.05).

**Table 2:** Durations of labor

	total VBAC group (*n* = 139)	VBAC group A (*n* = 109)	VBAC group B (*n* = 30)	Primipara Control group (*n* = 149)	Multipara Control group (*n* = 155)	*P* values				
						*p*^*a*^	*p*^*b*^	*p*^*c*^	*p*^*d*^	*p*^*e*^
The first stage of labor (min)	390 (240, 660)	405 (242, 657)	304 (240, 770)	630 (377, 847.5)	380 (260, 530)	0.001	0.378	0.001	0.764	0.411
The second stage of labor (min)	39 (15, 83)	39 (14, 77)	48 (26, 98)	72 (42, 113.5)	15 (9, 43)	0.001	0.001	0.001	0.001	0.206
The third stage of labor (min)	5 (4, 7)	5 (4, 7)	5 (3, 6)	5 (4, 8)	6 (5, 10)	0.378	0.001	0.671	0.002	0.283
The total stage of labor (min)	453 (267, 739)	479 (268, 733)	404 (264, 801)	703 (472, 941.5)	435 (280, 600)	0.001	0.230	0.001	0.895	0.610

The comparisons of durations of labor were without normal distribution and were conducted by Mann–Whitney U test

Data are given as median (interquartile range, IQR)

^a^ The comparison between total VBAC group and Primipara Control group

^b^ The comparison between total VBAC group and Multipara Control group

^c^ The comparison between VBAC group A and Primipara Control group

^d^ The comparison between VBAC group B and Multipara Control group

^e^ The comparison between VBAC group A and VBAC group B

### Intrapartum interventions

As shown in Table 3, women who had a successful VBAC were more likely to require mediolateral episiotomy, which showed a statistical difference between the total VBAC group and primipara control group (39 (28.1%) vs. 11 (7.4%), *P*=0.001), between the VBAC group A and primipara control group (29 (26.6%) vs. 11 (7.4%), *P*=0.001), between the total VBAC group and the multipara control group (39 (28.1%) vs. 9 (5.8%), *P*=0.001), and between the VBAC group B and the multipara control group (10 (33.3%) vs. 9 (5.8%), *P*=0.001). But there was no significant difference in the rates of forceps delivery among these groups (*P* > 0.05).

**Table 3:** Usage of interventions during the labor

	total VBAC group (*n* = 139)	VBAC group A (*n* = 109)	VBAC group B (*n* = 30)	Primipara Control group (*n* = 149)	Multipara Control group (*n* = 155)	*P* values				
						*p*^a^	*p*^b^	*p*^c^	*p*^d^	*p*^e^
Artificial membrane breaking	29 (20.9)	23 (21.1)	6 (20.0)	50 (33.6)	20 (12.9)	0.016	0.067	0.000	0.461	0.895
Oxytocin usage	16 (11.5)	11 (10.1)	5 (16.7)	71 (47.7)	15 (9.7)	0.001	0.609	0.001	0.432	0.499
Labor analgesia	60 (43.2)	49 (45.0)	11 (36.7)	110 (73.8)	64 (41.3)	0.001	0.745	0.001	0.637	0.417
phloroglucinol	8 (5.8)	8 (7.3)	0 (0.0)	17 (11.4)	1 (0.6)	0.089	0.028	0.275	1.000	0.278
Mediolateral episiotomy	39 (28.1)	29 (26.6)	10 (33.3)	11 (7.4)	9 (5.8)	0.001	0.001	0.001	0.001	0.534
Forceps delivery	5 (3.6)	4 (3.7)	1 (3.3)	4 (2.7)	1 (0.6)	0.916	0.169	0.652	0.299	1.000

Data are given as n (%).

^a^ The comparison between total VBAC group and Primipara Control group.

^b^ The comparison between total VBAC group and Multipara Control group.

^c^ The comparison between VBAC group A and Primipara Control group.

^d^ The comparison between VBAC group B and Multipara Control group.

^e^ The comparison between VBAC group A and VBAC group B.

The rates of oxytocin usage and labor analgesia in the total VBAC group and VBAC group A were lower than those in the primipara control group (*P* <0.05). Similar results were showed in the rate of AROM (*P* <0.05). The rate of phloroglucinol usage was higher in the total VBAC group than that in the multipara control group (*P* <0.05), and it did not differ between the total VBAC group and the primipara control group.

There were no significant differences in the rates of AROM, oxytocin usage, labor analgesia, phloroglucinol usage, mediolateral episiotomy and forceps delivery between VBAC group A and VBAC group B (*P* > 0.05).

### Maternal and neonatal outcomes of the study groups

Neonates in all groups had similar Apgar scores at 1-minute and 5-minutes (*P*>0.05), and there was no neonatal asphyxia in each group. There was no significant difference in the incidence of postpartum urinary retention among all groups (*P*>0.05, Table 4).

**Table 4:** Maternal and neonatal outcomes of the study groups

	total VBAC group (*n* = 139)	VBAC group A (*n* = 109)	VBAC group B (*n* = 30)	Primipara Control group (*n* = 149)	Multipara Control group (*n* = 155)	*P* values				
						*p*^a^	*p*^b^	*p*^c^	*p*^d^	*p*^e^
Apgar score at 1 min	10 (10, 10)	10 (10, 10)	10 (10, 10)	10 (10, 10)	10 (10, 10)	0.102	0.138	0.245	0.915	0.261
Apgar score at 5 min	10 (10, 10)	10 (10, 10)	10 (10, 10)	10 (10, 10)	10 (10, 10)	0.142	0.743	0.098	0.444	0.456
Blood loss during birth and 2 h postpartum (ml)	210 (140, 410)	200 (140,365)	310 (167,472)	190 (140,250)	140 (110,195)	0.012	0.001	0.077	0.001	0.091
Postpartum hemorrhage	19 (13.7)	14 (12.8)	5 (16.7)	8 (5.4)	5 (3.2)	0.016	0.001	0.034	0.011	0.462
Fever during labor	17 (12.2)	15 (13.8)	2 (6.7)	11 (7.4)	3 (1.9)	0.165	0.001	0.093	0.186	0.349
Perineal laceration	86 (86.0)	67 (83.3)	19 (95.0)	93 (67.4)	120 (82.2)	0.001	0.427	0.008	0.146	0.195
Postpartum urinary retention	5 (3.6)	4 (3.7)	1 (3.3)	7 (4.7)	1 (0.6)	0.640	0.169	0.686	0.299	1.000

Data are given as n (%) or median (interquartile range, IQR)

^a^ The comparison between total VBAC group and Primipara Control group

^b^ The comparison between total VBAC group and Multipara Control group

^c^ The comparison between VBAC group A and Primipara Control group

^d^ The comparison between VBAC group B and Multipara Control group

^e^ The comparison between VBAC group A and VBAC group B

No differences in the rates of perineal laceration were found between the total VBAC group and the multipara control group, between the VBAC group B and the multipara control group, and between VBAC group A and VBAC group B (*P* > 0.05), but it was higher in total VBAC group than primipara control group (86% vs. 67.4%, *P*=0.001). Similarly, the rate of perineal laceration in the VBAC group A was also higher than that in the primipara control group (83.8% vs. 67.4%, *P*=0.001).

Blood loss during birth and 2 hours postpartum and the incidence of PPH in women who had a VBAC were higher than those in primipara or multipara women (*P* <0.05), except that the mothers in the VBAC group A had a non-significant increased risk of blood loss during birth and 2 hours postpartum (*P*=0.077). However, they did not differ between VBAC group A and VBAC group B (*P* > 0.05).

The incidence of fever during labor in the total VBAC group did not differ from that in the primipara control group (*P* > 0.05), but was higher than that in the multipara control group (17 (12.2%) vs. 3 (1.9%), *P* =0.001). There was no significant difference in the incidence of fever during labor between the VBAC group A and the primipara control group, between the VBAC group B and the multipara control group, and between the VBAC group A and B(*P* > 0.05).

## Discussion

It was the first time to find that labor characteristics, intrapartum interventions and perinatal outcomes in VBAC women with cervical dilation strongly resembled those in VBAC women without cervical dilation before the previous cesarean section, but differed significantly from those of multiparae and primiparae.

This study found that labor characteristics, intrapartum interventions and perinatal outcomes in women who had a VBAC with cervical dilation were similar to those in women who had a VBAC without cervical dilation before the previous cesarean section, but differed significantly from those of multiparae and primiparae who not undergoing VBAC.

### The durations of labor

We observed that women who underwent VBAC had shorter first and total stage of labor than primiparous women not undergoing VBAC, but were comparable to the multiparous women not undergoing VBAC. Likewise, Zdenek Rusavy et al. showed women with VBAC had a shorter first stage of labor than primiparous women [11]. However, Grylka-Baeschlin et al. demonstrated that overall and first-stage labor duration in women with VBAC were comparable to that in primiparae but significantly longer than that in multiparae [8]. The conflicting results may be due to the differences in the study design, the sample size, the heterogeneous study population, as well as intrapartum usage of oxytocin and analgesia [12, 13]. Prospective, multicenter, large-scale trials are needed to elucidate the characteristics of labor in women with VBAC.

Considerable attention had been paid to the durations of labor in women who had a VBAC with and without cervical dilation in their prior labor. We found women who had a VBAC with cervical dilation showed comparable first and total stage of labor than multiparae. However, compared with primiparae, we found women who had a VBAC without cervical dilation showed shorter first and total stage of labor. The reduced cervical resistance to dilatation in parous women might account for the differences [11].

As for the second stage of labor, we found it was shorter in women who had a VBAC than that in primiparous women, but longer than that in multiparous women, which was similar to the findings of a previous study [8]. Likewise, women who underwent VBAC without cervical dilation showed a shorter second stage of labor than primiparae, while those with cervical dilation showed a longer one than multiparae. It might be related to the loss of pelvic floor contractility in prior pregnancy [14]. Limited research exists regarding the third stage of labor, and we found that the third stage of labor was shorter for women with VBAC than for multiparae. Similar results were discovered between women who had a VBAC with cervical dilation and multiparae. However, no difference in the third stage of labor was found between women with VBAC and primiparous women, and between women who had a VBAC without cervical dilation and primiparous women. This might be because our midwives paid more attention to the women with VBAC and took more active measures to prevent the occurrence of PPH, resulting in early delivery of the placenta in the third stage of labor.

### Usage of interventions during the labor

Oxytocin usage and AROM are routine methods to strengthen contractions and accelerate labor whenever required, which are associated with increased rates of uterine rupture during VBAC [5, 13]. Given the dose-dependent relationship between oxytocin use and uterine rupture [13], low-dose oxytocin could be safe and effective in VBAC. Our study analyzed the rate of oxytocin usage and the rate of AROM among women with VBAC, primiparae and multiparae. The results showed that the rate of oxytocin and AROM usage in women who had a VBAC was lower than those in primiparae but comparable to those in multiparae, which was consistent with a previous study [15]. Grylka-Baeschlin et al. also found the women with VBAC received oxytocin significantly less often than primiparae, but more often than multiparae [8]. This may be attributed to different demographic characteristics, including ethnicity and genetic profile, and gestational age. Therefore, oxytocin was less administrated to women who had a VBAC compared to the primiparous women not multiparous women.

Phloroglucinol is recommended to facilitate labor not only by reducing spasms and edema of the cervix but also by harmonizing shrinkage of the uterus [9]. Besides, Tabassum et al. found that pain intensity seemed lower in laboring women who received phloroglucinol as compared to those who received placebo [10]. This might be because pain during the birth mainly comes from dilation of the cervix and contraction of the uterus. Phloroglucinol, as one of the spasmolytics and spasmoanalgesics, also showed few side effects in both mother and fetus [9, 10]. In our study, the women with VBAC were more likely to use phloroglucinol than multiparae, but not primiparae. Furthermore, the rate of phloroglucinol usage in the women who had a VBAC with cervical dilation before the previous cesarean section was similar to that of multiparae, while the rate of phloroglucinol usage in the women who had a VBAC without cervical dilation before the previous cesarean section was similar to that of primiparae, which might be because they have similar cervical conditions.

The fear of masking the pain of a uterine rupture had made the use of epidural anesthesia a dilemma [5]. However, epidural analgesia is encouraged for women undergoing planned VBAC to provide pain control without increasing the risk of postpartum bleeding or uterine rupture [16]. Our data showed women with successful VBAC had a lower rate of epidural analgesia than primiparae but was comparable to multiparae, which was similar to a former research [15]. The rate of epidural analgesia might be related to different durations of labor in women between groups.

Some studies demonstrated prolonged labour, especially the prolongation in the second stage of labor, was associated with multiple adverse maternal and foetal outcomes such as obstructed labour, postpartum haemorrhage, perineal injuries [4, 9, 17]. To shorten the second stage of labor, episiotomy and forceps were frequently used for assisted vaginal birth [13]. Our study showed that episiotomy was more common for women who had a VBAC compared with multiparous women and primiparous women, which was similar to a recent study [2]. Shortening the second stage of labor to avoid uterine rupture may be responsible for the increased use of episiotomy in women who had a VBAC. However, Zdenek Rusavy et al. found that primiparous women and multiparous women not undergoing VBAC had comparable rates of episiotomy to women with VBAC [11]. The differences may be explained by differences in the discretion of obstetricians and indications for episiotomy. 

There were no significant differences in the rates of forceps deliveries among women who had a VBAC, primiparae and multiparae in our study. And Madi JM et al. [18] found the rate of forceps deliveries was 5.3% in women who underwent VBAC, which was similar to our result (3.6%). Forceps-assisted vaginal births were associated with maternal adverse outcomes such as sphincter damage, pudendal nerve damage, third- and fourth-degree perineal laceration, as well as neonatal adverse outcomes like subdural or cerebral hemorrhage, facial-nerve injury, brachial plexus injury, and the increased rate of mechanical ventilation [14, 17, 19, 20]. Consequently, the use of forceps should be minimized whenever possible.

### Maternal and neonatal outcomes of the study groups

Previous studies had shown a positive correlation between perineal lacerations and assisted vaginal birth, but most of these studies concentrated on third- and fourth-degree perineal tears [3, 11, 20, 21]. Our study included perineal lacerations from first- to fourth-degree, and the result was consistent with the observation reported elsewhere [11], showing a higher risk of spontaneous perineal tears in women who had a VBAC, especially in those without cervical dilation when compared to the primipara control group. One possible explanation is that the faster progress of labor in women who had a VBAC without cervical dilation, combined with nulliparous pelvic floor, may lead to a higher risk of perineal rupture [11, 19].

PPH can be caused primarily by atony uterus, retained tissue, genital tract tear, coagulation problem, and uterine rupture [22]. Previous studies had almost focused on the rate of PPH in VBAC and elective repeat cesarean birth, and some of them found PPH occurred more often in VBAC and mothers with PPH were exposed to more blood transfusion [13, 22, 23], but others revealed VBAC was associated with a lower incidence of PPH and was considerably less expensive than repeat cesarean section [2–4]. It was the first time, as far as the authors were aware, that the rates of PPH between women who underwent VBAC and women not undergoing VBAC who gave birth vaginally were compared. Our study showed a higher rate of PPH in women who had a VBAC than in primiparae and mulriparae. We also found there was more blood loss during and after VBAC within two hours than primiparae and multiparae delivered by vaginal. That’s probably because, with the increase of parity and gravidity, women’s myometrial muscular strength may get reduced due to the reduction of collagen fibers, especially in women with a history of cesarean section [22]. Therefore, we should be alert to the occurrence of PPH during VBAC.

 With regard to infectious complications, maternal fever was more common in VBAC than elective repeat cesarean birth [6]. Rita E. Fisler et al. found the increased rate of maternal intrapartum fever was associated with the use of epidural analgesia, resulting in adverse neonatal outcomes [24]. However, the relationship between epidural analgesia and the rate of maternal intrapartum fever in our study was not clear. Limited data exists in comparing the rate of maternal fever between women with VBAC and those with vaginal birth not undergoing VBAC. Our research found no differences between women who underwent VBAC without cervix dilation before the previous cesarean section and primiparae, women who underwent VBAC with cervix dilation and multiparae, women who underwent VBAC and primiparae. However, intrapartum fever occurred more often in women with VBAC than multiparae, which might be the result of the prolonged labor. Few previous studies have investigated postpartum urinary retention in women who had a VBAC. It was the first time to find there was no significant difference in the occurrence of postpartum urinary retention among women with VBAC, primiparae and multiparae. Regarding the postnatal condition of the newborn such as neonatal asphyxia, Apgar score in 1st minute plays an important role. No significant difference was found in women who had normal spontaneous vaginal birth as compared to women who had a VBAC [2], which was similar to our study. A previous study indicated that compared with a trial of labor, there was a higher rate of transient tachypnea of the newborn after elective repeat cesarean section [24]. Therefore, VBAC may reduce the occurrence of neonatal asphyxia.

### Strengths and limitations

The major strength of the present study lies in its design. Unlike most previous studies on this topic, our study took cervical dilation prior to the cesarean section into account. It was the first time to compare durations of labor, intrapartum interventions and perinatal outcomes between women who had a VBAC without cervical dilation before the previous cesarean section and primiparae, and between women who had a VBAC with cervical dilation before the previous cesarean section and multiparae, respectively. Besides, we found a remarkable resemblance in labor characteristics, intrapartum interventions, and perinatal outcomes between women who had a VBAC with cervical dilation before the previous cesarean section and those without cervical dilation. Therefore, planned VBAC is well recommended in women without contraindications of VBAC. The major limitation of the study is certainly the number of women in our groups, however, the size still allowed a proper statistical analysis. 

## Conclusions

In conclusion, labor characteristics, intrapartum interventions and perinatal outcomes in women who had a VBAC with cervix dilation strongly were similar to those in women who had a VBAC without cervix dilation before the previous cesarean section, but differed significantly from those of multiparae and primiparae who did not undergo VBAC. Women with VBAC had shorter first and total labor than primiparae, but comparable to multiparae, and showed a longer second stage of labor than primiparae, but shorter than multiparae. The duration of the third stage of labor was shorter than both primiparae and multiparae. AROM, oxytocin, and epidural analgesia were less used for women with VBAC versus primiparas. Phloroglucinol was more used for women with VBAC versus multiparas. Women who had a VBAC were more likely to receive episiotomy and had higher incidences of PPH than primipara and multipara women. Therefore, strict labor management, especially the management of the second stage labor in women who had a VBAC, should be emphasized.

## Supplementary Information

**Additional file 1.**

## Data Availability

All data generated or analysed during this study are included in this published article [and its supplementary information files].

## Abbreviations

VBAC: Vaginal birth after cesarean section;
CS: Cesarean section
RCS: Repeated cesarean section
BMI: Body mass index
PPH: Postpartum hemorrhage
AROM: Artificial rupture of membrane
